# BMP Pathway Regulation of and by Macrophages

**DOI:** 10.1371/journal.pone.0094119

**Published:** 2014-04-08

**Authors:** Megha Talati, James West, Rinat Zaynagetdinov, Charles C. Hong, Wei Han, Tom Blackwell, Linda Robinson, Timothy S. Blackwell, Kirk Lane

**Affiliations:** 1 Department of Medicine, Vanderbilt University, Nashville, Tennessee, United States of America; 2 Department of Research Medicine, Veterans Affairs Tennessee Valley Healthcare System, Nashville, Tennessee, United States of America; University Medical Center of the Johannes Gutenberg University of Mainz, Germany

## Abstract

Pulmonary arterial hypertension (PAH) is a disease of progressively increasing pulmonary vascular resistance, associated with mutations of the type 2 receptor for the BMP pathway, BMPR2. The canonical signaling pathway for BMPR2 is through the SMAD family of transcription factors. BMPR2 is expressed in every cell type, but the impact of BMPR2 mutations affecting SMAD signaling, such as Bmpr2^delx4+^, had only previously been investigated in smooth muscle and endothelium. In the present study, we created a mouse with universal doxycycline-inducible expression of Bmpr2^delx4+^ in order to determine if broader expression had an impact relevant to the development of PAH. We found that the most obvious phenotype was a dramatic, but patchy, increase in pulmonary inflammation. We crossed these double transgenic mice onto an NF-κB reporter strain, and by luciferase assays on live mice, individual organs and isolated macrophages, we narrowed down the origin of the inflammatory phenotype to constitutive activation of tissue macrophages. Study of bone marrow-derived macrophages from mutant and wild-type mice suggested a baseline difference in differentiation state in Bmpr2 mutants. When activated with LPS, both mutant and wild-type macrophages secrete BMP pathway inhibitors sufficient to suppress BMP pathway activity in smooth muscle cells (SMC) treated with conditioned media. Functionally, co-culture with macrophages results in a BMP signaling-dependent increase in scratch closure in cultured SMC. We conclude that SMAD signaling through BMP is responsible, in part, for preventing macrophage activation in both live animals and in cells in culture, and that activated macrophages secrete BMP inhibitors in sufficient quantity to cause paracrine effect on vascular smooth muscle.

## Introduction

Pulmonary arterial hypertension (PAH) is a disease characterized by progressively increasing pulmonary vascular resistance, leading to right heart failure and death. Mutations in the type 2 receptor for the bone morphogenic protein (BMP) pathway, BMPR2, are responsible for the majority of the heritable form of pulmonary arterial hypertension [1], [2], and sizable minority of the idiopathic form [3]. The BMP pathway appears to be suppressed in PAH even where BMPR2 mutation is not involved [4], [5]. Studies of the effect of BMPR2 mutation in adult lung are complicated by fetal death in knockouts [6] and a subtle phenotype in heterozygote BMPR2 knockouts [7].

To overcome this, our group has over-expressed dominantly acting Bmpr2 mutations under the control of doxycycline sensitive promoters in a tissue specific manner. This allows us to bypass the developmental difficulties, but still drive the mutation strongly enough to create a distinctive phenotype in adults. Bmpr2 signals through at least two direct mechanisms; a kinase domain, which regulates the SMAD transcription factors, and a cytoplasmic tail which regulates cytoskeletal functions [8]. We have previously shown that the Bmpr2^delx4+^ mutation, which truncates the receptor just after the transmembrane domain, acts as a dominant negative for SMAD signaling and causes PAH, secondary to smooth muscle dedifferentiation, when expressed only in smooth muscle [9], [10]. It also causes PAH, secondary to inflammatory and clotting difficulties, when expressed only in endothelium [11]. However, we have never previously expressed the Bmpr2^delx4+^ mutation in all tissue types, and the initial motivation for the current study was simply to determine whether universal expression had a phenotype distinct from smooth muscle- or endothelium- specific expression.

We were surprised to find that the phenotype of universal Bmpr2^delx4+^ mutation, while it included mild PAH, was dominated by inflammation not just of the pulmonary vasculature, but also of airways. This inflammatory phenotype appeared to be driven by a primary defect in tissue macrophages, which had nuclear factor of kappa B (NF-κB) activation in the absence of stimulus. Follow-up studies in bone marrow derived macrophages (BMDM) showed that Bmpr2 mutant macrophages had mild constitutive, possibly classical, activation, and that activated wild-type macrophages secreted BMP inhibitors to an extent sufficient to alter BMP reporter and BMP-dependent behavior in smooth muscle cells with co-culture or conditioned media.

## Methods

### Rosa26-rtTA2 X TetO_7_-Bmpr2^delx4+^ Mice

For these studies, we bred our previously published Rosa26-rtTA2 mice [8] with our previously published Bmpr2^delx4+^ mice [10] to create mice heterozygous in both genes and expression of the mutations inducible with doxycycline, in chow. Both strains are on an FVB/N background, and mice were used in early adulthood (aged 12–14 weeks at sacrifice).

### Immunohistochemistry & Quantitation of Septa

Hematoxylin and eosin-stained paraffin sections from Rosa26-Bmpr2^delx4+^ mice were analyzed by morphometry (magnification X 400). Thickness of the interalveolar septa (IAS) (L_alv_, μm) was measured sequentially for 20 septa in each of the 10 randomly chosen fields and measured by calibrated image analysis using Image-Pro Express (Media Cybernetics, Silver Springs, MD). Immunohistochemistry on paraffin embedded lung tissue for Rosa26-Bmpr2^delx4+^ included the following antibodies: CD45 (sc25590 Santa Cruz), and CD11b (BD Pharmingen 550282). Immunohistochemistry on archival paraffin embedded lung tissue from HPAH patient or control (rejected transplant lung) were done with antibody for RelA/p65 (sc-372 Santa Cruz).

### Flow Cytometry

Cells isolated from lung and spleen were used for analysis. Lungs were perfused until free of blood by visual inspection and digested in RPMI-1640 medium containing collagenase XI (0.7 mg/ml; Sigma-Aldrich, St. Louis, MO, USA) and type IV bovine pancreatic DNase (30 μg/ml; Sigma-Aldrich, St. Louis, MO, USA). To obtain single-cell suspensions, lungs and spleens were passed through 70 μm BD Falcon cell strainer, followed by treatment of cell aggregates with RBC Lysis Buffer (BioLegend, San Diego, CA, USA) and staining using Abs: CD4-PE and CD11b-APC-Cy7 from Abcam, CD8-Pacific Blue from Biolegend, CD34 Alexa 647, CD45-Alexa Fluor 700 (30-F11), MHCII-Biotin (Clone NIMR-4) from eBioscience, CD86 PE-Cy7 (GL1) from BD Bioscience and F4/80-PE (Clone BM8) from Invitrogen (Carlsbad, CA, USA). Flow cytometry was performed using BD LSR II flow cytometer (BD Bioscience, San Diego, CA, USA) and data were analyzed with FlowJo software (TreeStar, Ashland, OR, USA).

### Rosa26-rtTA2 X TetO_7_-Bmpr2^delx4+^ X HLL or NGL Mice

HLL and NGL mice are previously described NF-κB reporter strains that express luciferase under the control of an NF-κB dependent promoter [12]. These were bred to Rosa26-Bmpr2^delx4+^ mice to make Rosa26/HLL or Rosa26-Bmpr2^delx4+^/HLL double and triple transgenic mice. Adult mice were fed doxycycline in chow and NF-κB -dependent luciferase activity was measured, as previously described [13], on days 2, 4, 14, 21, and 35. Briefly, mice were anesthetized, chest and abdomen shaved, injected with Luciferin, and imaged under an intensified charge-coupled device. This is a survival procedure: the same mice are used at each time point. At the end of week 5, mice were sacrificed and organs collected for measurement of luciferase activity. All animal procedures were approved by the Vanderbilt Institutional Animal Care and Use Committee (IACUC).

### Derivation of Macrophages

Macrophages were derived from the bone marrow of the mouse femur. The skin around the area of the femur was soaked with 70% ETOH. The femur was exposed, using a scalpel, removed, and scraped to clean away all tissue. It was then placed in a Kimwipe® tissue soaked with 70% ETOH and rubbed to remove any remaining tissue. Using sterile scissors and tweezers, the ends of the femur were removed. The interior of the femur was flushed with a 25-gauge needle, containing 3 mls PBS, into a 15 ml conical tube. The tube was centrifuged for five minutes at 120 RCF, the cells re-suspended in bone marrow-derived macrophage media (BMDM: 10% FBS, 10% L929 conditioned media, 1% pen/strep, 1% L-glutamine) and allowed to mature for five days.

Macrophages were derived by this method from wild type and transgenic mice, including: double-transgenic for Rosa26-rtTA2 and TetO7-BMPR2^delx4+^ (allowing doxycycline-inducible expression of the delx4+ BMPR2 mutation); triple transgenic for Rosa26-rtTA2, TetO7-BMPR2^delx4+^ and an NFkB-responsive promoter (driving luciferase [14]); transgenic for only the NFkB-responsive promoter. To avoid doxycycline specific effects, all macrophage cultures were maintained in 300 ng/ml doxycycline. All animal procedures were approved by the Vanderbilt Institutional Animal Care and Use Committee.

### Time Course

An average of 1.5×10^6^ macrophages were derived from six wild type and six BMPR2^delx4+^ mice. 2.5×10^5^ macrophages from each mouse were plated in duplicate and incubated overnight in DMEM, 10% FBS, and 1% pen/strep+1% l-glutamine and 300 ng/ml doxycycline. The next day, treatments included: 100 ng/ml LPS with harvest after 2 and 4 hours; 100 ng/ml LPS with LPS removed after 4 hours, then harvested at 24 hours; vehicle only. For each time point, macrophage conditioned media was removed and stored at −80°C for the conditioned media experiment below.

### Cytokine Array Panel

Bone marrow derived macrophages from five wild type and five BMPR2^delx4+^ mice were harvested as above. 2.5×10^5^ macrophages from each mouse were plated in each well of a 6 well plates and incubated overnight in DMEM, 10% FBS, and 1% pen/strep+1% l-glutamine and 300 ng/ml doxycycline. The next day, cells were treated with 100 ng/ml LPS or vehicle for 4 hours, resulting in three wells per condition per mouse, and five mice per genotype. Cells were washed with media containing Polymyxin B sulfate to remove the LPS, and cell supernatants harvested after 24 hours. Supernatants from each of the four conditions (+/− mutation, +/− LPS) were pooled before application to the arrays, to reduce variability. R&D systems mouse cytokine antibody arrays (Catalog # ARY006) were used according to the manufacturer’s instructions.

### Quantitative RT-PCR

RNA was purified from macrophages using an RNEasy kit (Qiagen) according to the manufacturer’s instructions.

First strand cDNA was made from 1 μg total RNA using a QuantiTect® Reverse Transcription Kit (Qiagen, Valencia, CA). Quantitative real-time PCR was performed using iTaq Power SYBR Green (Applied Biosystems, Warrington, UK) in a StepOnePlus Real Time PCR System (Applied Biosystems, Foster City, CA), (95°C-10 min, [95°C (15 sec), 60°C (1 min), 40 cycles]. Each measurement was made in triplicate and expressed relative to the detection of the standard HPRT. Primer sets are detailed in Table 1.

**Table 1:** Primer sets.

	Forward	Reverse	Product
Grem1	CATACACTGTGGGAGCGTTG	TCATTGTGCTGAGCCTTGTC	115 bp
Grem2	CAACTCCTTCTACATCCCGC	TTCTTGATTCGGAAAGGTGG	141 bp
Hprt	TGCTCGAGATGTCATGAAGGAG	TTTAATGTAATCCAGCAGGTCAGC	103 bp
Id1	GTGAGCAAGGTGGAGATCCTG	GGTGGTCCCGACTTCAGACTC	90 bp
Mac1	CTTCTGGTCACAGCCCTAGC	ACCACACTCTGTCCAAAGCC	101 bp
Mrc1	AACAAGAATGGTGGGCAGTC	TTTGCAAAGTTGGGTTCTCC	128 bp
Smad6	ACCAACTCCCTCATCACTGC	TGGTCGTACACCGCATAGAG	140 bp

### Conditioned Media Experiment

A confluent T175 flask of A7r5 vascular smooth muscle cells was lifted with trypsin and plated onto two p100 plates in DMEM with 10% FBS, 1% pen/strep, 1% l-glutamine, for overnight incubation. Cells were transfected with BRE-Luciferase [15] (pBRE-Luc) and TK Renilla (Promega, Madison WI)(as control) using Superfect transfection reagent and protocol (Gibco #301305). After incubation for two hours with the lipid/plasmid mix, cells were lifted with trypsin, counted, and replated in 24 well plates at 75,000 cells per well. After overnight incubation, treatments included: conditioned media from resting macrophages; macrophages activated by 4 hours of LPS treatment, with 50 ng/ml recombinant BMP4; 100 ng/ml LPS. All conditions were tested in triplicate; BMP4 and LPS were pre-incubated for one hour with supernatants. After four hours incubation, wells were washed and lysed for dual luciferase assay.

### Co-Culture

A7r5 smooth muscle cells were transfected with pBRE-Luc and TK Renilla, as above, then plated onto 6-well plates at 250,000 cells/well and allowed to recover overnight. Macrophages were plated on top of CO-STAR inserts (at 500,000 cells/well) in separate six well plates and incubated overnight. For activation, macrophages were treated for four hours with LPS at 100 ng/ml and were washed twice with PBS to remove LPS before being added to the A7r5 plates. Resting and activated macrophage CO-STAR inserts were placed above A7r5 cells in 6-well plates overnight with some wells receiving 50 nM DM-3189 BMPR1 receptor inhibitor [16]. After 24 hours, A7r5 cells were lysed for dual luciferase assay.

### Scratch Assay

The wild-type (WT) BMPR2 expression construct was created by integrating a full-length human cDNA for BMPR2 into pciNEO. Mutated forms were created using the QuikChange Site-Directed Mutagenesis Kit (Stratagene). These included construct F14, with a T354G mutation resulting in C118W; construct KD, with a C993T mutation resulting in R332X; and construct CD, with a 2579–2580delT resulting in a frameshift at amino acid 859 resulting in 10 missense amino acids and a stop. All of these mutations are copies of mutations found in human PAH patient families. A7r5 cells were stably transfected with constructs described above, by antibiotic selection. Expression arrays for these cells have been previously published [17].

A7r5 cells either un-transfected or stably transfected, with WT, F14, KD, or CD constructs, were grown to confluence and then scratched with a P-1000 pipette tip. A dot was placed on the underside near the center of the plate on the scratch, and pictures were taken of the scratch with the dot just out of field at 0 and 48 hours. Macrophage co-culture used CO-STAR inserts to physically separate them from the A7r5 vascular smooth muscle cells, as per co-culture experiments above. Experiments were performed in triplicate. Scratch healing was quantified by taking an area of remaining field, divided by the length of the scratch visible, to correct for irregularities in healing and in the angle at which the scratch was photographed, resulting in an average width measurement.

Although pulmonary artery smooth muscle cells would have been preferable, the need for stable transfections required use of an immortalized line, and A7r5 were the only closely related immortalized line available. These cells, stably transfected with BMPR2 mutations, have been extensively characterized previously [17].

### Statistical Methods

ANOVA or multiple ANOVA was used for most figures, with post-hoc Fisher’s least significant difference (LSD) tests, and correlation z-test used for time-course data, using statistical software JMP (SAS Institute, Cary, NC).

## Results

### Rosa26-Bmpr2^Delx4+^ Mice have Mild Pulmonary Hypertension

**Figure 1:**
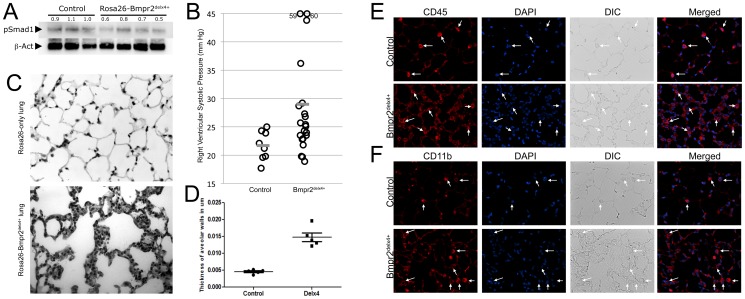
Universal expression of a Bmpr2 mutation that impacts Smad signaling results in a pulmonary inflammatory phenotype (A) Rosa26-Bmpr2^delx4+^ transgenic mice have a decrease in BMP pathway activity of about 40%, as assessed by canonical BMP phosphorylation target Smad1 (B) Rosa26-Bmpr2^delx4+^ transgenic mice develop mild pulmonary arterial hypertension after 8 weeks of doxycycline induction in adult mice. Each circle is an individual mouse. Significant at p = .0124 by t-test and.025 by Wilcoxon. Grey bars indicate mean RVSP. (**C**) Lung from Rosa26-Bmpr2^delx4+^ mice includes patches with dramatic alveolar inflammation. (**D**) Rosa26-Bmpr2^delx4+^ mice have a near tripling of septal thickness accompanying this inflammation. Each point represents the average of 10 measurements in each of 10 fields in a particular mouse. (**E**) Lungs from Rosa26-Bmpr2^delx4+^ transgenic mice have a large increase in CD45+ circulating leukocytes (red) and (**F**) CD11b+ macrophages (red).

We crossed our transgenic mouse expressing the reverse tetracycline trans-activator (rtTA2) in all tissues [8] to our mouse with doxycycline-inducible expression of the Bmpr2 mutation with kinase domain truncation, Bmpr2^delx4+^ [10]. When fed doxycycline, these mice express, in all tissues, the Bmpr2^delx4+^ mutation, which blocks both canonical SMAD signaling as well as cytoskeletal tail domain signaling (**Figure 1A**).

The experiment included 24 Rosa26-Bmpr2^delx4+^ and 10 Rosa26-only control mice. Starting at approximately 8 weeks old (adult), doxycycline was fed in chow for 8 weeks. We then measured systemic pressures by tail cuff, and right ventricular systolic pressure (RVSP) by closed-chested intra-jugular cardiac catheterization. Groups had no difference in systemic systolic pressures: controls averaged 95 mm Hg and Bmpr2^delx4+^ averaged 96 mm Hg (not shown). Both average and median RVSP in control mice were 21.7 mm Hg, while Bmpr2^delx4+^ mice had an average of 29.0 and a median of 25.1 mm Hg, (**Figure 1B**).

Because we had previously published a decrease in markers in smooth muscle differentiation in smooth muscle-specific expression of the Bmpr2^delx4+^ transgene [9], we measured expression of smooth muscle differentiation markers calponin 1 (CNN1) and titan (TTN). In keeping with our previous publication, we found them reduced on average in Rosa26-Bmpr2^delx4+^ mice as compared to controls by 26% and 59%, respectively.

### Rosa26-Bmpr2^Delx4+^ Mice have Strong Pulmonary Inflammation

While Bmpr2^delx4+^ mice had pulmonary hypertension, it was mild (<20% with RVSP over 30 mm Hg). The dominant phenotype appeared to consist of pulmonary inflammation (**Figure 1C**), including a near tripling of average septal thickness (**Figure 1D**). This phenotype was patchy within individual mice: some regions appeared near normal, while ∼10%–30% of the area of each lung, varying from mouse to mouse, was strongly inflamed. Immunohistochemical staining showed an increase in the presence of inflammatory cells in the lung (**Figure 1E**), and in particular CD11b positive macrophages (**Figure 1F**).

**Figure 2:**
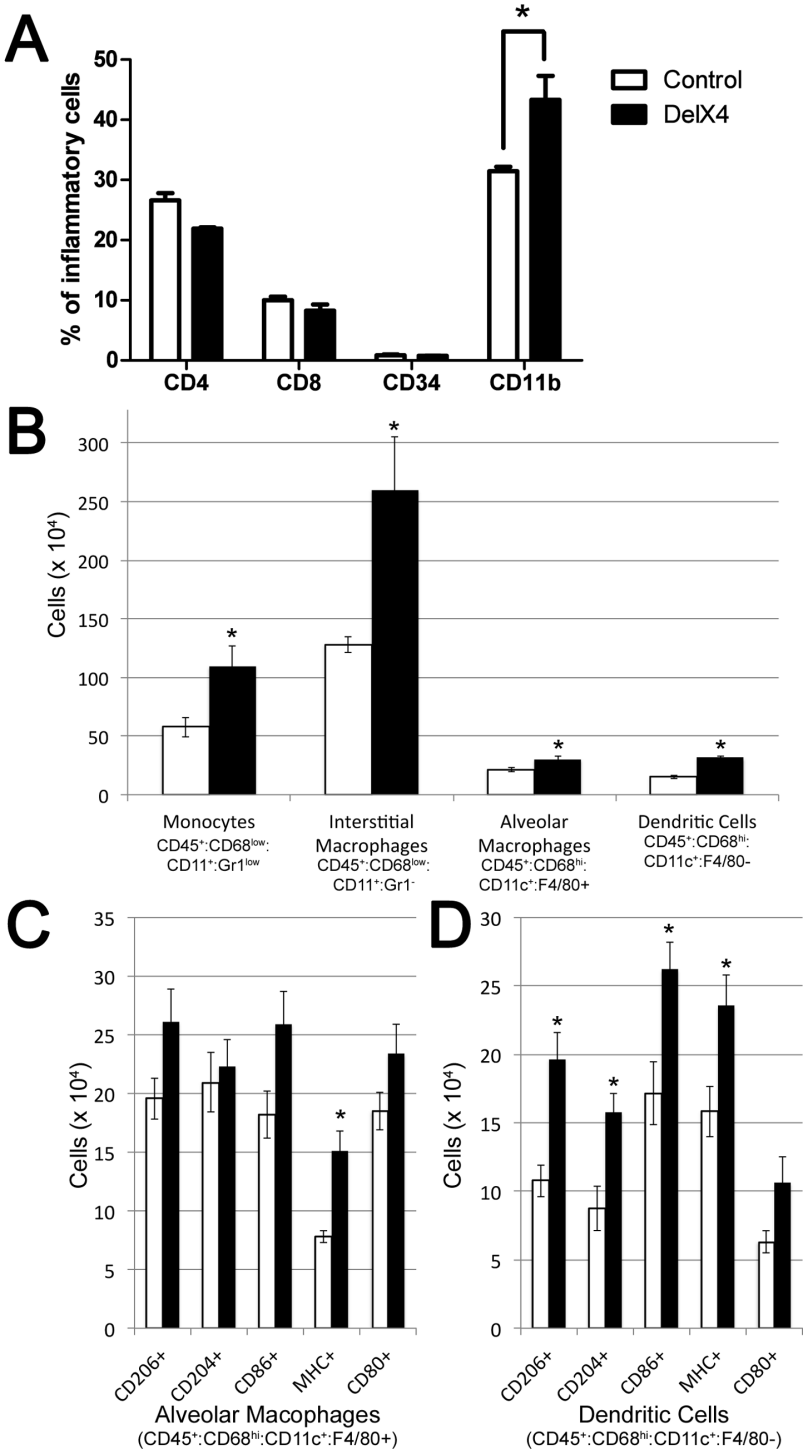
The increased inflammatory cells in lungs from Bmpr2^delx4+^ transgenic mice are primarily in the monocyte lineage. (**A**) Number of CD11b cells as a proportion of CD45+ leukocytes in the lung is increased in Rosa26-Bmpr2^delx4+^ mice (p<.01 by ANOVA). (**B**) All categories of CD68 positive circulating cells showed significant increases (* = p<.05), with the increase numerically dominated by interstitial macrophages. (**C & D**) Some subtypes of alveolar macrophages and dendritic cells also show statistically significant increases (* = p<.05).

Flow sorting of whole homogenized lung, gated on CD45+ cells, demonstrated that the proportion of CD11b macrophages in the lung had increased relative to other types of inflammatory cells (**Figure 2A**).

CD11b+ macrophages are tissue or interstitial macrophages, not alveolar macrophages, which are CD11b− [18]. To narrow down the subtypes of monocytic cells which were increased, we performed flow sorting on an additional 4 control and 4 Rosa26-Bmpr2^delx4+^ mice with additional markers. We found that most categories of monocytic lineages were increased, but with the increase numerically dominated by monocytes and interstitial macrophages (**Figure 2B, C, D**).

### Rosa26-Bmpr2^Delx4+^ Mice have Spleen-specific NF-κB Activation

**Figure 3:**
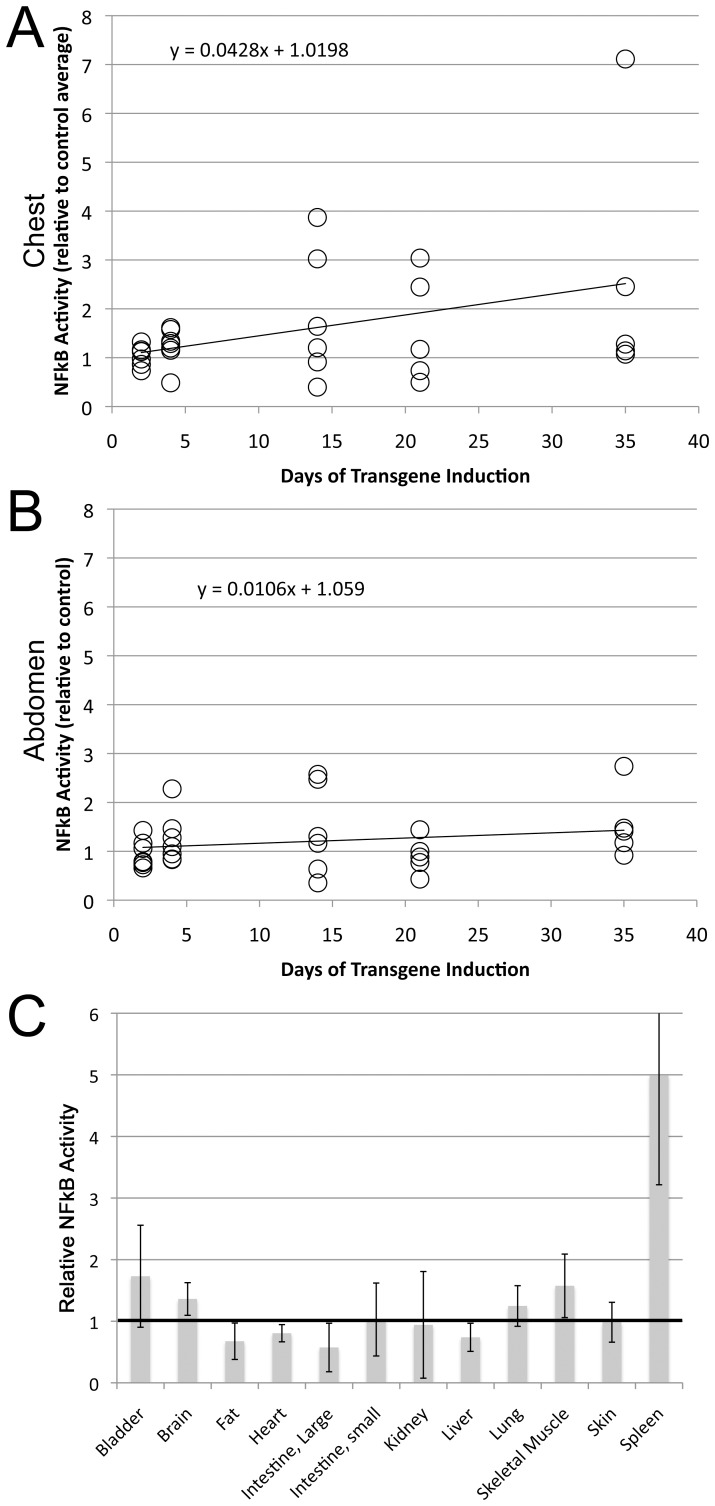
Although Bmpr2^delx4+^ causes increased NF-κB activity, this is probably the result of inflammatory cell recruitment (A) Bmpr2^delx4+^ causes increased NF-κB activity in the chest. P = .03 by correlation z-test, p<.01 by multiple ANOVA for genotype effect, Fischer’s LSD shows 14 days different than controls at p<.01, 35 days different than controls at p<.05. Each circle corresponds to an individual animal, normalized to control measurements. (**B**) Bmpr2^delx4+^ does not cause increased NF-κB activity in the abdomen by either correlation z-test or multiple ANOVA. (**C**) NF-κB is increased ∼5x in spleen from Bmpr2^delx4+^ transgenic mice (p<.05 by ANOVA), but not significantly in other organs (luciferase activity is normalized to that in controls, n = 4–8 per organ per group).

To determine whether this pulmonary inflammatory phenotype was related to NF-κB activation, we crossed the Rosa26-Bmpr2^delx4+^ mice onto the NF-κB luciferase reporter strain, NGL [12]. Adult triple-transgenic mice had transgene Bmpr2^delx4+^ induced with doxycycline, and 11 control (NGL X Rosa26) and 7 triple transgenic (NGL X Rosa26-Bmpr2^delx4+^) had luciferase measured at repeated time-points over 5 weeks. We found that luciferase activity in the chest increased significantly over time (**Figure 3A**), but luciferase activity in the abdomen did not (**Figure 3B**). Note that the extinction distance for the light through mouse tissue is quite short, and so only the top few millimeters is likely being assayed through in vivo measurements. At 35 days, animals were sacrificed, and organs collected for ex-vivo measurement of organ-specific NF-κB driven luciferase activity. We found that only the spleen showed a significant increase in NF-κB activity (**Figure 3C**). The lack of significance in lung as an organ, given the in vivo data, probably arises from two factors: first, significance in the chest came from correlation z-test, which allows use of multiple time points to achieve significance that would not have been present by t-test. Second, at the 35 day time point, the only time point used in assessment of organs, NF-κB activity in chest trends lower in most animals than it had been at 2 and 3 weeks.

### Splenic Macrophages from Rosa26-Bmpr2^Delx4+^ Mice are Constitutively Active

**Figure 4:**
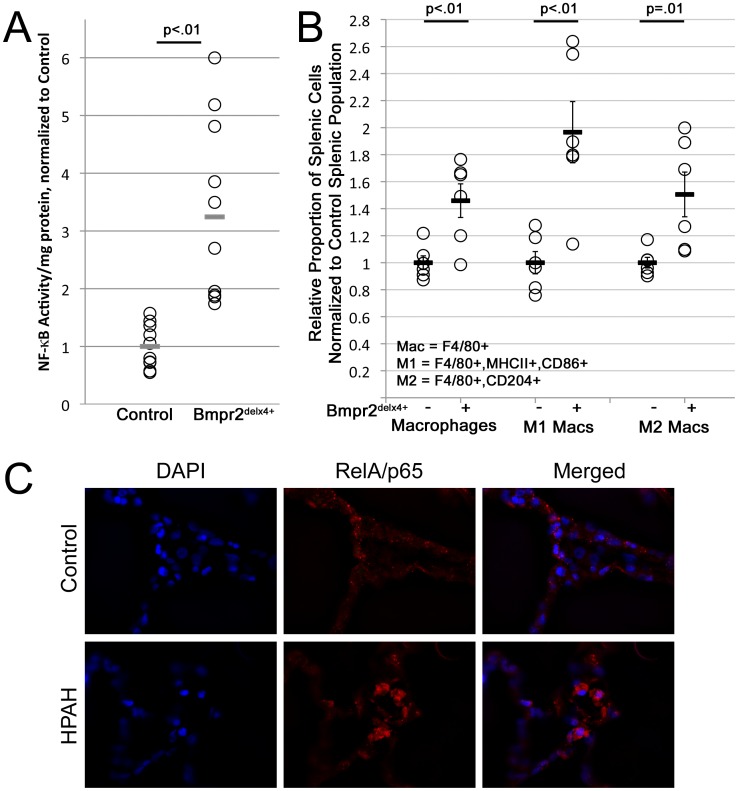
Splenic macrophage number is increased and NF-κB activity is tripled in Bmpr2^delx4+^ mice. (**A**) Freshly isolated splenic macrophages from Bmpr2^delx4+^ mice have increased NF-κB activity, p = .0002 by t-test or Wilcoxon. (**B**) Bmpr2^delx4+^ mice have increased proportion of spleen macrophages, both overall and M1 or M2 polarized (p<.01, <.01, = .01 by ANOVA, respectively.) (**C**) Immunohistochemistry in lungs from control (top) and heritable PAH patients (bottom) suggests that the majority of NF-κB activity is in HPAH patients is in cells which, morphologically, appear to be of circulating origin.

NF-κB activity, only increased in spleen, suggests that its primary effect was in monocytes or macrophages. To test this, we bred ten HLL X (NF-κB luciferase reporter) Rosa26 control mice, and eight triple-transgenic HLL X Rosa26-Bmpr2^delx4+^ mice. After two weeks of transgenic activation with doxycycline chow, spleens underwent FACS sorting for CD11b+ macrophages, and levels of NF-κB activation were determined by luciferase reporter assay. We found that splenic macrophages had an average of more than threefold increased NF-κB activation (**Figure 4A**) coupled with increased numbers of both M1- and M2- polarized splenic macrophages expressing MHC II and CD86 or CD204 (scavenger receptor A), respectively (**Figure 4B**).

The combination of this data with data from figures 2 and 3 suggests that CD11b+ tissue macrophages in all tissues have increased NF-κB activation; it is more visible in spleen than in other organs because of the very high numbers of tissue macrophages in spleen compared to other organs. To determine whether this is consistent with human PAH, we performed immunohistochemistry on control lung and lung derived from a heritable PAH (HPAH) patient. We found that NF-κB activation in human HPAH lung appears localized to inflammatory cells (**Figure 4C**). This is in agreement with both older publications [19], [20], and with a recent comprehensive analysis of NF-κB activation in macrophages in end-stage human PAH [21].

### Bone Marrow Derived Macrophages from Rosa26-Bmpr2^Delx4+^ Mice Show Constitutive Activation

**Figure 5:**
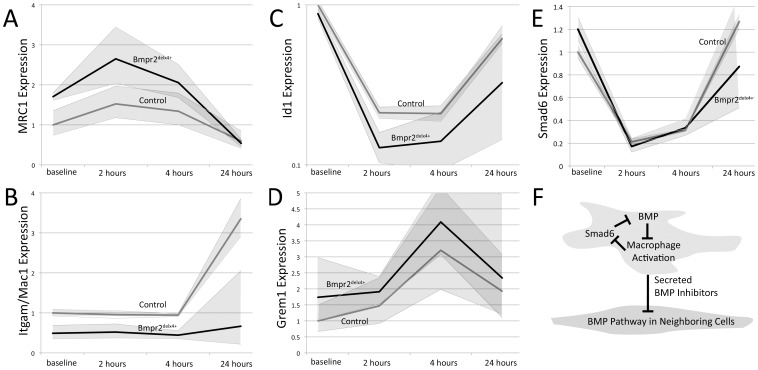
Gene expression alterations in bone marrow-derived macrophages (BMDM) from Bmpr2^delx4+^ mice compared to controls. Shaded areas indicate SEM; all results are normalized to HPRT expression and then to control baseline. (**A**) Classical activation marker MRC1 is increased in BMDM from Bmpr2^delx4+^ mice. (**B**) Alternative activation marker Itgam is decreased in BMDM from Bmpr2^delx4+^ mice. (**C**) Canonical BMP pathway target Id1 is strongly suppressed by inflammation. (**D**) Secreted BMP pathway inhibitor Grem1 is induced by inflammation. (**E**) Intracellular BMP pathway inhibitor Smad6 is suppressed by inflammation. (**F**) In combination, these suggest that the BMP pathway normally suppresses classical macrophage activation; when macrophages are activated, they produce secreted factors to suppress the BMP pathway and intracellular factors to reduce suppression of the pathway.

To further investigate Bmpr2-mediated changes in macrophage function, we collected bone marrow derived macrophages (BMDM) from wild-type and Bmpr2^delx4+^ mice, treated them with LPS, and measured indicators of inflammatory and BMP pathway response at baseline, 2, 4, and 24 hours. Macrophages from four mice per genotype were used, with three experimental replicates per macrophage line. IL-6 activity was strongly (∼10,000x) induced in all cells with LPS treatment, confirming uniform activation starting from ∼40% higher expression levels in mutants (not shown). We found that Bmpr2^delx4+^ macrophages appeared to have a different baseline activation state, with nearly twofold increase in activation of classical activation marker and PU.1 target, MRC1 (**Figure 5A**), but a twofold decrease in alternative activation marker Mac1/Itgam, which failed to reliably increase in late stage in mutants (**Figure 5B**). Sfpi1, which encodes PU.1, was not different between controls and mutants (not shown). Canonical BMP signaling target ID1 is moderately lower at baseline in Bmpr2^delx4+^ macrophages, and is strongly suppressed in both control (fivefold below normal) and mutant macrophages (eightfold below normal) after LPS treatment (**Figure 5C**). This pattern was similar for Id3 (not shown). Expression of secreted BMP inhibitor Grem1 was induced, on average 3–4x, in both mutant and control cells (**Figure 5D**). Grem2 has roughly the same pattern, with a ∼3x induction at 4 hours (p = .029 by multiple ANOVA) (not shown). In contrast, intracellular BMP pathway inhibitor Smad6 is roughly 5x reduced at 2 hours in both control and mutant macrophages (**Figure 5E**). In contrast, the intracellular TGFβ pathway inhibitor Smad7 is induced by approximately 60% (1.6x) in both control and mutant macrophages. In combination, these data suggest a feedback loop in which intracellular signaling attempts to restore the BMP pathway, loss of which correlates with activation, while extracellular signaling suppresses BMP pathway activation in nearby cells (**Figure 5F**).

### Bone Marrow Derived Macrophages from Rosa26-Bmpr2^Delx4+^ Mice Show Increased Cytokine Secretion when Activated

**Figure 6:**
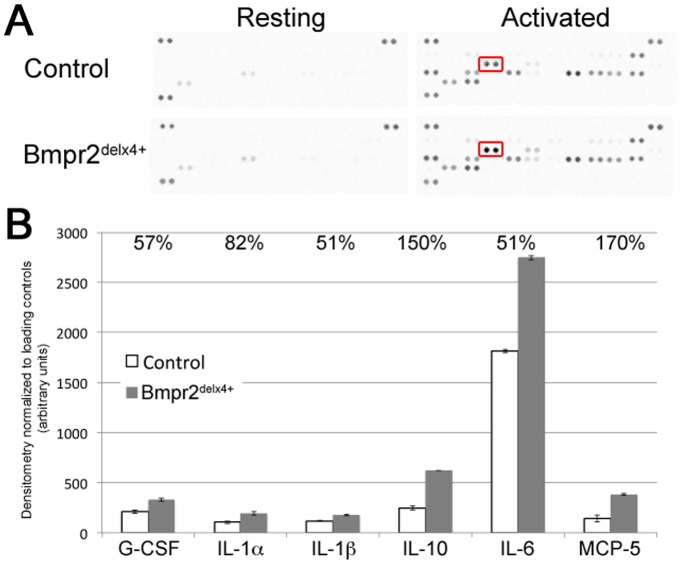
Pooled supernatants show increased cytokine production in activated bone-marrow derived macrophages from 5 independent Bmpr2^delx4+^ mice. (**A**) Cytokine antibody arrays; each pair of dots is an independent antibody, with 40 cytokines depicted per array. Spots in the top left and right and bottom left are used for normalization. The set representing interleukin 6 (IL-6) is boxed. (**B**) Normalized densitometry shows increases in interleukins 1α, 1β, 6, and 10, granulocyte colony stimulating factor (G-CSF), and monocyte chemoattractant protein 5 (MCP-5). All changes are significant at p<.01 by ANOVA.

To determine whether this moderate increase in activation state markers resulted in an increase in cytokine secretion, macrophage supernatant was applied to cytokine antibody arrays. Pooled supernatant from five different control bone marrow derived macrophage and five different Bmpr2^delx4+^ macrophages, with and without LPS, were applied to separate R&D systems mouse cytokine arrays (**Figure 6A**). In supernatant from resting macrophages, there were only two measurable cytokines: tissue inhibitor of metalloproteinase 1, and monocyte chemoattractant protein 1. Neither were significantly altered in Bmpr2 mutants compared to controls.

In activated macrophages, however, of 40 cytokines measured, six were upregulated more than 50% in Bmpr2^delx4+^ macrophages as compared to controls (**Figure 6B**). These included interleukins 1α, 1β, 6, and 10, granulocyte colony stimulating factor (G-CSF), and monocyte chemoattractant protein 5 (MCP-5). The strongest absolute change was in interleukin 6 (IL-6), previously reported to be induced in Bmpr2 mutants [22].

### Macrophage Conditioned Media Suppresses the BMP Pathway in Vascular Smooth Muscle Cells

**Figure 7:**
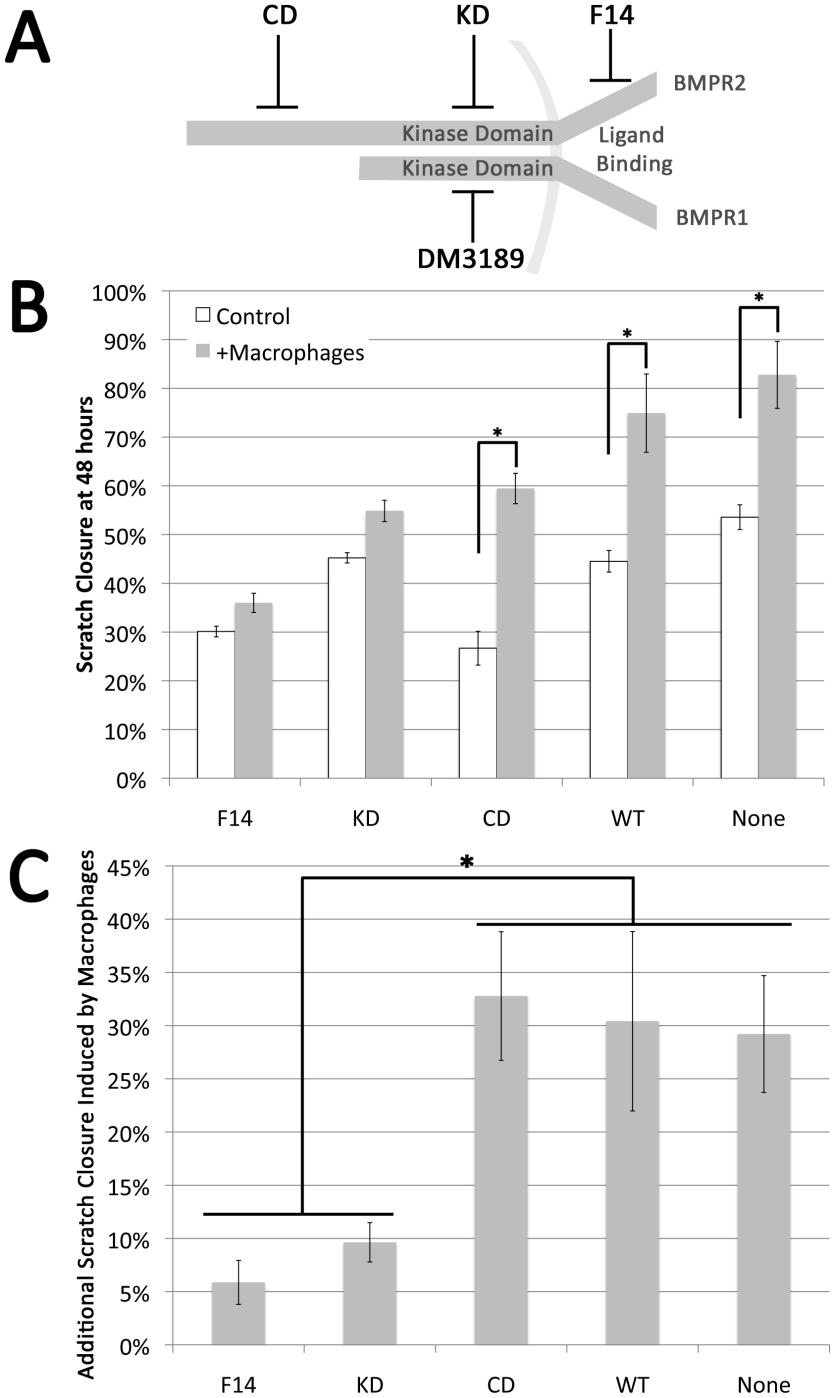
Macrophages induce increased scratch closure in smooth muscle in a manner dependent on intact BMPR2 receptor (A) Macrophage conditioned media inhibits BMP pathway activity in A7r5 vascular smooth muscle cells by BMP response element-luciferase reporter assay. Multiple ANOVA reported a p<0.0001 for comparison between macrophage treated and untreated cells, and a trend towards additional inhibition by BMPR2 mutant macrophages. * = p<.05 for comparison shown by post-hoc t-test. (**B**) A7r5 coculture with macrophages inhibits BMP pathway activity, not cumulative with BMPRI inhibition by DM-3189. Significance was determined by two-way ANOVA. * = p<.05 for difference between control and macrophage conditioned media. § = p<.05 for difference between level of DM-3189 inhibition with stimulated macrophages and with either control or resting macrophages.

While two secreted BMP inhibitors, Grem1 and Grem2, showed up-regulation in response to macrophage activation, we wished to determine whether this resulted in signaling consequences. We thus tested the effects of macrophage-conditioned media on BMP pathway activity in a vascular smooth muscle cell line, A7r5, by pBRE-Luc assay. We found that media with 10% FBS strongly activates the BMP pathway, and the pathway was down regulated roughly 2.5x by macrophage conditioning, regardless of BMP mutation or activation state (**Figure 7A**). The addition of 50 ng/ml BMP increased signal in non-conditioned media by about 50%. It was still significantly inhibited by macrophage conditioning, with a trend (p = .08) to increased inhibition in media conditioned by BMPR2^delx4+^ macrophages.

To confirm this finding using a different system, and in order to examine mutual signaling and receptor specificity, A7r5 vascular smooth muscle cells transfected with pBRE-Luc were co-cultured with either resting wild-type macrophages or stimulated wild-type macrophages (with LPS removed shortly before co-culturing). Some cells also received the specific BMP type I receptor inhibitor DM-3189 [16]. The purpose of DM-3189 was to establish that the effect was mediated through BMP receptors, rather than some process internal to A7r5 cells mediated by non-BMP signaling between macrophage and smooth muscle cells. We found nearly identical results; either resting or stimulated macrophages suppressed BMP pathway activity about 2.5x compared to control, requiring type I receptor for effect, although type I receptor inhibitors could suppress BMP pathway activity further (**Figure 7B**). In the presence of DM-3189, stimulated macrophages suppressed BMP pathway activity slightly, but significantly, beyond that seen with either no-macrophage control or resting macrophages, suggesting that there is some impact on mechanisms not mediated by the BMP type I receptor, but that it is a trivial component of effect.

### Macrophage Co-culture Increases Scratch Closure in Vascular Smooth Muscle Cells, Dependent on SMAD-domain Signaling Through Bmpr2

**Figure 8:**
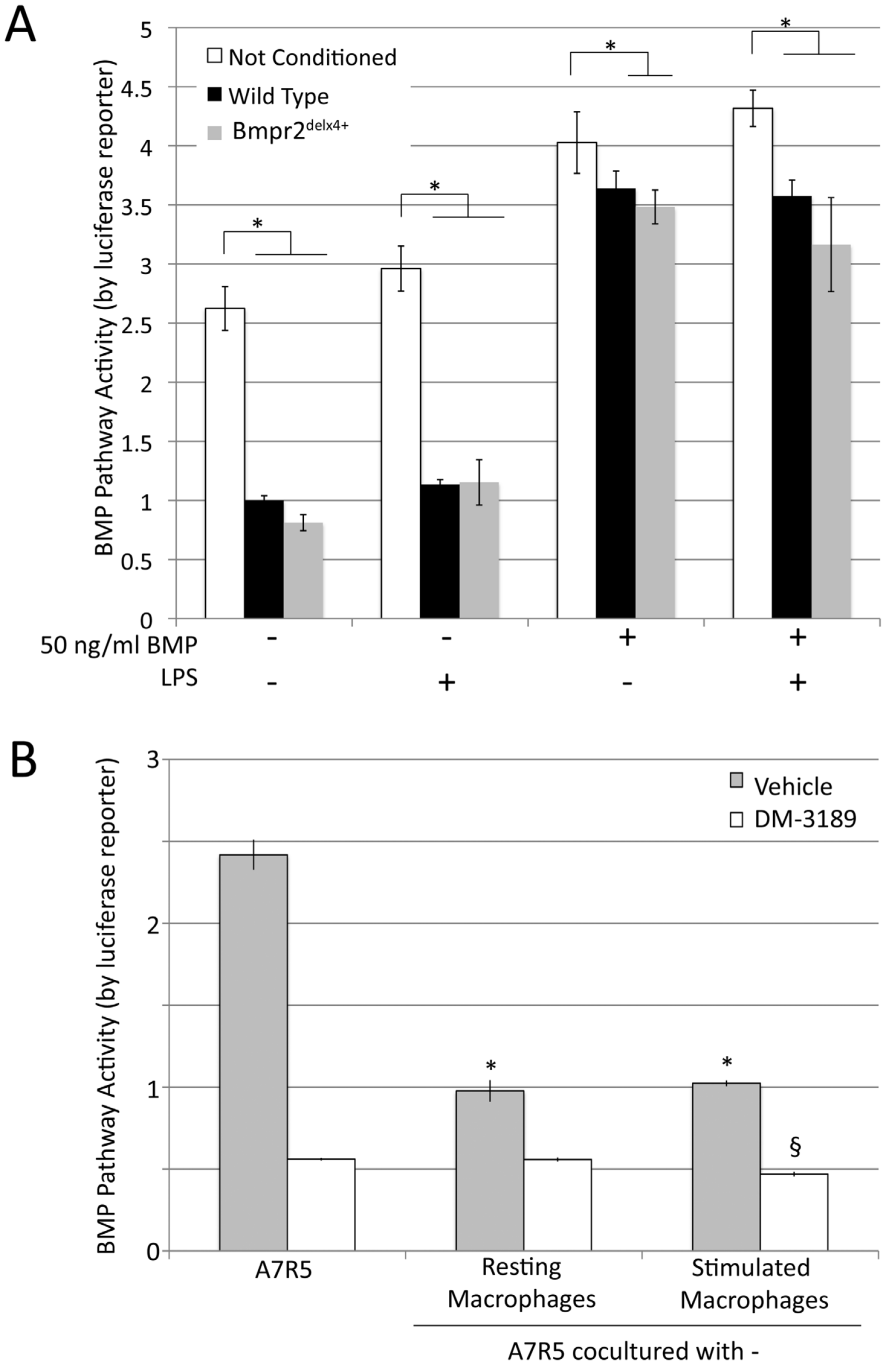
Interaction of soluble macrophage derived factors and smooth muscle growth. (**A**) Schematic representation of the BMP receptor complex and the location of BMPR2 mutations and the site of action of the BMPR1-SMAD signaling inhibitor DM3189. (**B**) Confluent cultures of A7r5 VSMC or A7r5 cells stably expressing human BMPR2 alleles that interfere with ligand binding (F14), truncate the protein in the kinase domain (KD) or truncate the cytoplasmic tail (CD) were wounded by drawing a pipette across the center of the culture. Companion wells were similarly wounded and co-cultured with macrophages on insert and the area of the exposed plastic quantitated. Data presented as the percent of closure at 48 hr in the absence (white bars) or presence of macrophages (shaded bars) for each cell line. (**C**) Quantification of effect of macrophage co-culture on A7r5 cells expressing listed BMPR2 alleles. Cells defective in ligand binding or truncated in the kinase domain fail to respond to macrophage co-culture (F14 and KD) cells with intact kinase domain remain macrophage responsive and respond similarly even in the presence of a cytoplasmic tail truncation.(CD, WT and A7r5 VSMC). Both presence of macrophages and constructs were significant by two-way ANOVA at p<.01; *indicates p<.01 by post-hoc t-test.

Our next goal was to determine whether paracrine inhibition of BMP signal in A7R5 vascular smooth muscle cells by macrophages had functional consequences in a scratch closure test. Because macrophages also secrete many cytokines in addition to BMP inhibitors (eg, **Figure 6**), specificity of effect for BMP inhibition was tested by using smooth muscle cells stably transfected with BMPR2 mutations. If effect is blocked by BMPR2 mutation it implies that the effect is caused by signal through BMPR2. We used A7r5 vascular smooth muscle cells stably transfected with either nothing, wild-type BMPR2 (WT, as a control), or BMPR2 with mutations in the ligand binding domain (F14), the kinase domain (KD), or the cytoplasmic tail domain (CD) (**Figure 8A**).

We found that co-culture with macrophages caused a significant increase in scratch closure in un-transfected A7r5 and A7r5 transfected with either wild type or cytoplasmic domain mutant BMPR2 (**Figure 8B**). However, cells transfected with mutated BMPR2, either ligand binding or kinase domain mutations, had very little increase in growth in response to macrophages (**Figure 8c**). This implies that induction of scratch closure caused by macrophages requires intact signaling through the BMPR2 kinase domain and that functional BMPR2 cytoplasmic tail domain is not important for this effect.

## Discussion

In this study, we found that the dominant pulmonary phenotype caused by suppression of SMAD signaling, through BMPR2 in all cell types in Rosa26-Bmpr2^delx4+^ mice, was strong inflammation in the airways associated with increased recruitment of macrophages (**Figures 1** **,** **2**). By crossing the Rosa26-Bmpr2^delx4+^ mice onto two different NF-κB reporter lines and examining effects in whole animal, tissue, and cells, we were able to narrow the source of NF-κB-dependent inflammation in Bmpr2^delx4+^ mice to constitutive activation of tissue macrophages (**Figures 3** **,** **4**). Comparing bone-marrow derived macrophages (BMDM) from control and Rosa26-Bmpr2^delx4+^ mice, we found that Bmpr2^delx4+^ led to increased markers of activation at baseline, but that both control and mutant BMDM secreted BMP inhibitor Gremlin 1, with increased expression on activation (**Figure 5**). Using either conditioned media or co-culture, macrophages suppressed BMP pathway signaling in vascular smooth muscle cells 2.5x (**Figure 7**). This was functionally meaningful: while macrophages normally increase rate of scratch closure in culture, this effect was dependent on suppression of BMP signaling through its kinase domain (**Figure 8**).

Taken as a whole, there are at least three important implications of this work.

First, the universal suppression of BMP signaling results in a pulmonary phenotype that doesn’t look like PAH (although there is associated PAH). The obvious epithelial phenotype is telling us that the BMP pathway is doing something important in the epithelia unrelated to PAH, but also that the suppression of BMPR2 in PAH is vascular-specific. We’ve previously shown that strong vascular-specific suppression of the BMP pathway using this mutation alone is sufficient to cause PAH [10], [11], but because we’re overexpressing a dominant negative, we’re driving pathway activity below that found with any human mutation alone. In patients with BMPR2 mutation, penetrance is modest and age of onset is highly variable, implying that the level of suppression in these patients is insufficient on its own to cause disease; the present data suggests that the second hit pushing BMPR2 expression lower must be vascular specific.

Secondly, this work implies the BMP pathway is important to macrophage activation. The fact that macrophages show both increased M1 and M2 populations (**Figure 2** **,** **4**) and fail to show increased alternative activation markers (**Figure 5B**), points to the possibility that these macrophages are inhibited in the ability to participate in the resolution of inflammation. This is particularly interesting in the context of PAH, in that decreased BMP signaling is a feature of PAH regardless of etiology [5], inflammation decreases BMP signaling in macrophages, and macrophages influence the growth and BMP signaling of vascular cells. If BMP inhibited macrophages are hindered in their ability to resolve inflammation, this failure to resolve inflammation may result in a negative feedback loop that spirals downward to further inhibit BMP signaling, thus worsening the PAH phenotype. A current weakness of the literature is that we have failed to distinguish between protective and pathologic inflammation: prostacyclin therapy, for instance, prolongs life, but appears to worsen inflammation [19]. There is a trend towards reduction in NF-κB activity in the chest in late stages in the mice (Figure 3A), but in this context, this is difficult to interpret.

A third, and perhaps most important, implication of this work is that a normal function of activated macrophages is to suppress the BMP pathway in a paracrine manner in their microenvironment. In the short term, this has effects beneficial to wound healing: it decreases endothelial cell-cell junctions [23] to improve macrophage invasion [19], and induces a metabolic shift [17], increased proliferation [24] and migration [25] to support wound healing. In normal injury, this is terminated by negative feedback signaling arising from both within the macrophage (eg via Smad6, **Fig. 5E**) and from the nearby vascular mural cells [11], [22]. Moreover, suppression of Smad6 not only acts as negative feedback, allowing reactivation of the BMP pathway, but also may directly disinhibit NF-κB activation [26]. Smad6 inhibition thus may be part of a short term activation and long term inhibition program. In PAH, either the presence of a BMPR2 mutation or, potentially, other genetic or environmental factors, results in continued suppression of the BMP pathway and a persistent inflammatory state.

Previous work has implicated maintenance of a persistent inflammatory state as potentially causative of human pulmonary arterial hypertension [27], [28]. Determination of the cause-effect relationship in human patients is complicated by the late stage of the disease and the high level of inflammatory activation associated with end-stage disease. However, in PAH associated with systemic sclerosis, macrophage expression of MRC1, as is induced by BMPR2 mutation in the present study, correlates perfectly with development of PAH [29]. Some of the most direct work done on the topic to date has shown that restoration of the anti-inflammatory effects of regulatory T-cells could prevent PAH in a rat model [29]. In sum, while this line of study is still in its early stages, existing publications support our hypothesis that failure to resolve macrophage activation may be at least partially causative of PAH.

The primary limitation to this study is one of stoichiometry: overexpression of a dominant negative Bmpr2 causes a more severe suppression of BMP signaling at baseline than is present in any human patient, where much of the suppression probably comes from additional environmental or genetic factors. These results are thus best used to indicate what can result from suppression of the BMP pathway, rather than as the inevitable result of typical BMPR2 mutations. Moreover, although the BMP pathway plays roles in almost every tissue type, because of our focus on finding the origin of NF-κB mediated inflammation, we have neglected its effect in other tissues and organs.

Related to this point, in **Figure 7**, macrophage paracrine suppression of BMP pathway activity was not dependent on BMP mutation status within the macrophages. However, inflammation in Rosa26-Bmpr2^delx4+^ mice was dramatically worsened, with strongly increased recruitment and activation in both lungs and spleen. Based on our previous work suggesting that BMPR2 mutation causes inflammatory feedback defects in smooth muscle directly [22], it seems likely that study in culture is failing to capture important long-term feedback loop effects. We also can’t distinguish between the effects of increased recruitment and increased activation with our current approach, although both are clearly present.

The central conclusions of this study are that BMPR2 plays a role in suppressing macrophage activation through SMAD signaling, and that even wild-type macrophages suppress BMP signaling in their microenvironment at functionally meaningful levels. These results are of potential importance to regulation of macrophage activation and function not just in pulmonary arterial hypertension, but potentially in any inflammatory process.
