# Correction: BMP Pathway Regulation of and by Macrophages

**DOI:** 10.1371/journal.pone.0101543

**Published:** 2014-06-23

**Authors:** The PLOS ONE Staff

The images for Figures 7 and 8 are incorrectly switched. The image that appears as Figure 7 should be Figure 8, and the image that appears as Figure 8 should be Figure 7. The figure legends appear in the correct order. The figures in their correct order can be viewed below.

**Figure 7:**
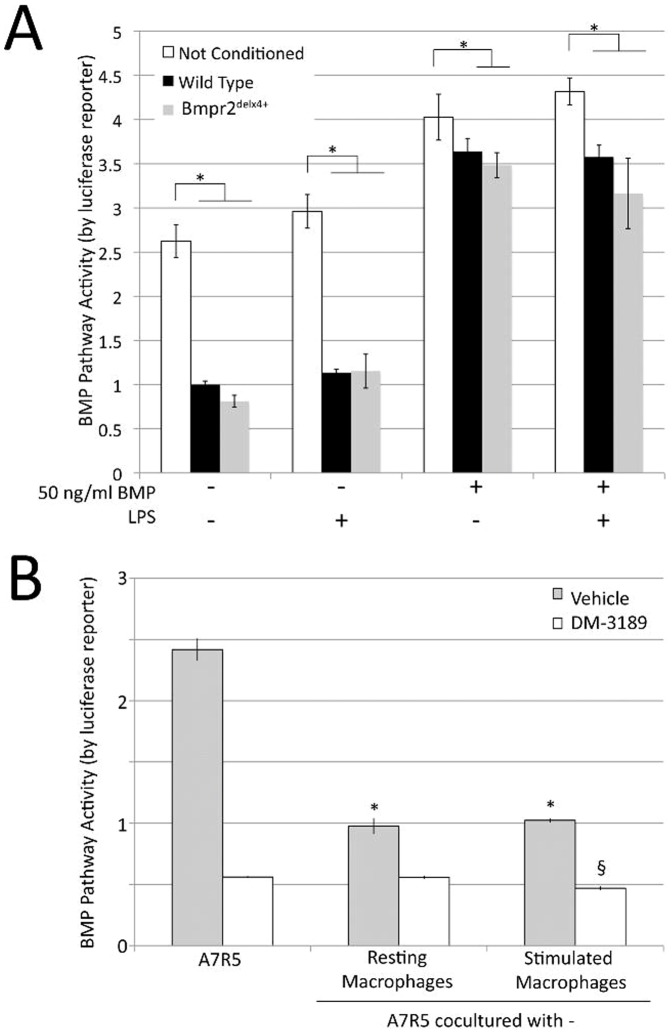
Macrophages induce increased scratch closure in smooth muscle in a manner dependent on intact BMPR2 receptor (A) Macrophage conditioned media inhibits BMP pathway activity in A7r5 vascular smooth muscle cells by BMP response element-luciferase reporter assay. Multiple ANOVA reported a p<0.0001 for comparison between macrophage treated and untreated cells, and a trend towards additional inhibition by BMPR2 mutant macrophages. *  =  p<.05 for comparison shown by post-hoc t-test. (**B**) A7r5 coculture with macrophages inhibits BMP pathway activity, not cumulative with BMPRI inhibition by DM-3189. Significance was determined by two-way ANOVA. *  =  p<.05 for difference between control and macrophage conditioned media. §  =  p<.05 for difference between level of DM-3189 inhibition with stimulated macrophages and with either control or resting macrophages.

**Figure 8:**
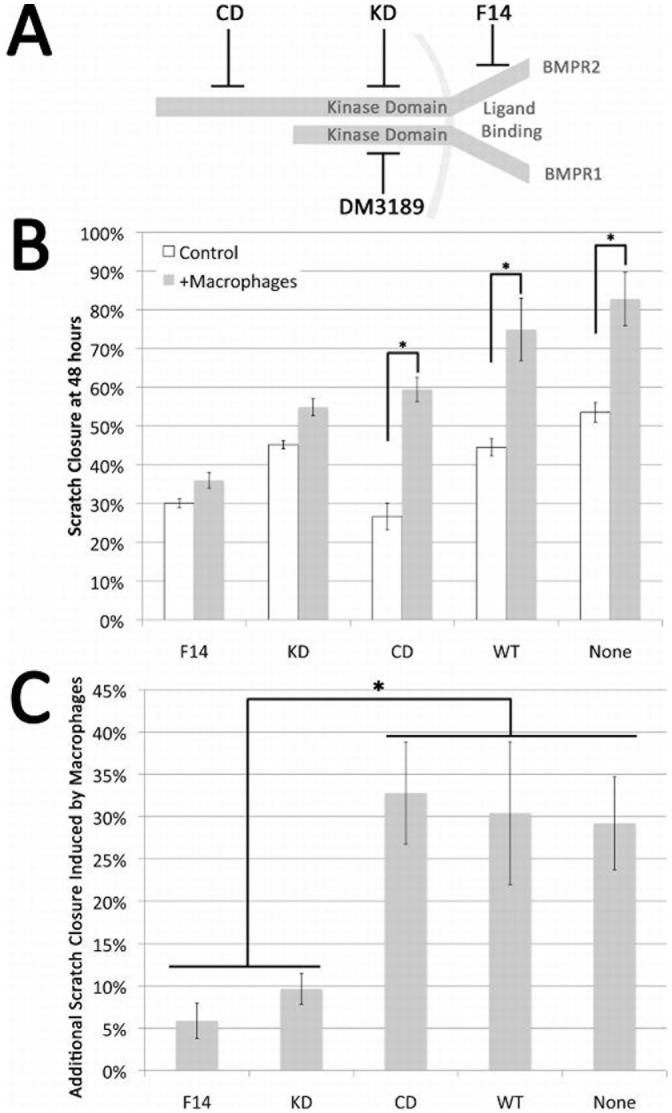
Interaction of soluble macrophage derived factors and smooth muscle growth. (**A**) Schematic representation of the BMP receptor complex and the location of BMPR2 mutations and the site of action of the BMPR1-SMAD signaling inhibitor DM3189. (**B**) Confluent cultures of A7r5 VSMC or A7r5 cells stably expressing human BMPR2 alleles that interfere with ligand binding (F14), truncate the protein in the kinase domain (KD) or truncate the cytoplasmic tail (CD) were wounded by drawing a pipette across the center of the culture. Companion wells were similarly wounded and co-cultured with macrophages on insert and the area of the exposed plastic quantitated. Data presented as the percent of closure at 48 hr in the absence (white bars) or presence of macrophages (shaded bars) for each cell line. (**C**) Quantification of effect of macrophage co-culture on A7r5 cells expressing listed BMPR2 alleles. Cells defective in ligand binding or truncated in the kinase domain fail to respond to macrophage co-culture (F14 and KD) cells with intact kinase domain remain macrophage responsive and respond similarly even in the presence of a cytoplasmic tail truncation.(CD, WT and A7r5 VSMC). Both presence of macrophages and constructs were significant by two-way ANOVA at p<.01; *indicates p<.01 by post-hoc t-test.
